# Message in a bottle: mRNA vaccination for influenza

**DOI:** 10.1099/jgv.0.001765

**Published:** 2022-07-13

**Authors:** Jessica R. Shartouny, Anice C. Lowen

**Affiliations:** ^1^​ Department of Microbiology and Immunology, Emory University School of Medicine, Atlanta, GA, USA; ^2^​ Emory Center of Excellence for Influenza Research and Response (Emory-CEIRR), USA

**Keywords:** influenza, mRNA vaccine, adjuvant, universal vaccine, strain selection, antigenic drift

## Abstract

Current influenza vaccines, while being the best method of managing viral outbreaks, have several major drawbacks that prevent them from being wholly-effective. They need to be updated regularly and require extensive resources to develop. When considering alternatives, the recent deployment of mRNA vaccines for SARS-CoV-2 has created a unique opportunity to evaluate a new platform for seasonal and pandemic influenza vaccines. The mRNA format has previously been examined for application to influenza and promising data suggest it may be a viable format for next-generation influenza vaccines. Here, we discuss the prospect of shifting global influenza vaccination efforts to an mRNA-based system that might allow better control over the product and immune responses and could aid in the development of a universal vaccine.

## Introduction: The rise of mRNA vaccination

Vaccines against influenza viruses have been used for more than 80 years but are still an active area of research and a major draw on public health resources as they need to be updated often to accommodate constantly-changing viruses [1]. The first vaccine for influenza was employed in the mid-1930s, influenza A viruses (IAVs) having just been discovered at Mill Hill a few years previously. Initially, immunization involved subcutaneous inoculation with formalin inactivated viruses grown in mouse lungs; soon after, egg-grown inactivated viruses were developed by Jonas Salk and Thomas Francis, Jr. for the United States military [2]. In the decades since, influenza vaccines have been expanded to include both IAVs and influenza B viruses (IBVs) and have become widely recommended for annual receipt. These vaccines are essential tools for controlling seasonal influenza and responding to influenza pandemics.

IAV and IBV, members of the Orthomyxoviridae family of negative-sense RNA viruses, are associated with respiratory disease in humans [3]. The influenza virus genome comprises eight segments and encodes ten essential proteins [4]. Influenza viruses are diverse and IAVs are subtyped based on the serological properties of their two surface glcyoproteins, hemagglutinin (HA) and neuraminidase (NA) [5, 6]. HA mediates entry into cells and receptor preferences of HA are a major determinant of viral species tropism [7]. NA releases newly-formed viruses from the originating cell by removing receptors from the cell surface [8]. Two distinct lineages of IBV, termed Yamagata and Victoria, and IAV of the H1N1 and H3N2 subtypes circulate in a sustained fashion in the global human population, causing recurrent outbreaks of respiratory illness [5]. Zoonotic transmission of IAV from birds or pigs occurs occasionally and can lead to pandemics, in which a novel IAV lineage is established in humans [9].

To allay seasonal illness, three types of influenza vaccines are used annually: inactivated, live-attenuated, and recombinant [10]. In all three platforms, the vaccine includes components from three or four influenza virus lineages that circulate endemically: one H1N1 subtype IAV, one H3N2 subtype IAV, and one or both of the Yamagata and Victoria IBV lineages [11]. Inactivated vaccines present the body with components of the viral envelope from killed viruses that are non-infectious [12]. These can be whole virus, split, where virions are disrupted by detergents, or subunit, where specific proteins are purified. Inactivated vaccines are delivered intramuscularly or intradermally and produce systemic immune responses. Live-attenuated vaccines (LAIV) contain replication-competent viruses that have cold-adapted and temperature-sensitive phenotypes and are thus maladapted to the temperature of the lower respiratory tract [13, 14]. LAIV are used in an intranasal format well-suited to eliciting mucosal immune responses at the major point of infection. For both inactivated vaccines and LAIV, the method developed in the 1930s of growing viruses in embryonated chicken eggs is still largely employed in production, though animal cell-based vaccines have become available more recently. Recombinant vaccine, made by producing HA in insect cells, is another more recent addition to the influenza vaccine market and is garnering approval in many countries [11].

Despite the current vaccination options, seasonal influenza viruses are estimated to cause several million illnesses and are associated with up to 650 000 deaths per year globally [15, 16]. Vaccine policies and coverage vary dramatically between countries: vaccination of those 65 or older ranged from less than 6 % of the population to more than 70 % in 2019 [17]. Even in countries with high vaccine uptake, seasonal influenza viruses impose a heavy disease burden that can be partially attributed to shortcomings of current vaccines [18]. The variability of the influenza virus surface antigens necessitates yearly vaccine administration to match currently-circulating strains; however, the time needed for vaccine production constrains efforts to respond to viral evolution in real time. Mismatching of the vaccine to prevalent strains can occur, leading to reduced vaccine effectiveness [19]. Even if matched well, the immunogenicity of current vaccines can be low in some high-risk groups, like older adults and those who are immunocompromised, who have diminished capacity to develop effective immune responses [20, 21]. For these reasons, improved vaccines that are safe, affordable, and elicit stronger, longer-lasting immunity is a key facet of the World Health Organization’s (WHO) Global Influenza Strategy to better control seasonal outbreaks [22].

**Fig. 1.**
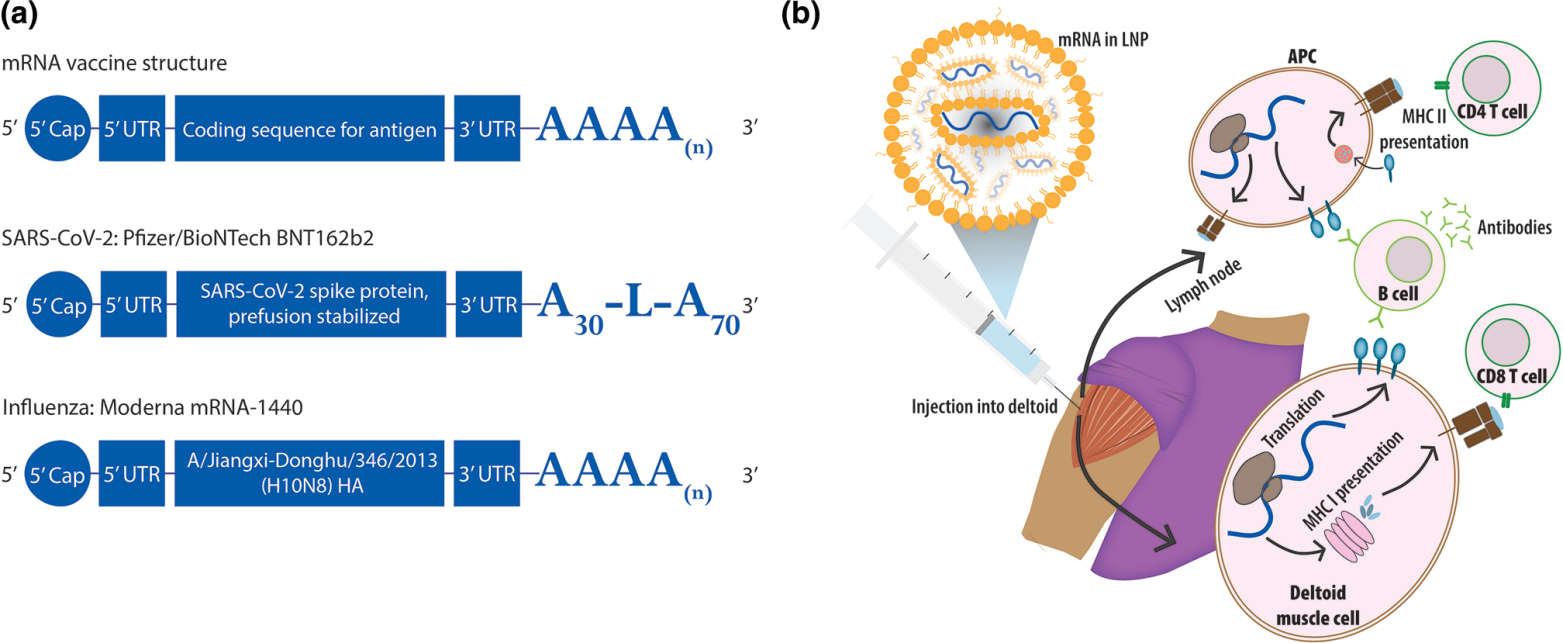
mRNA vaccine structure and function. (a) mRNA vaccines have multiple components, each of which can be optimized for administration: a 5’ cap, untranslated regions at the 5’ and 3’ ends, a coding region containing the antigens of choice, and a poly-A tail. The BNT162b2 SARS-CoV-2 mRNA vaccine contains the sequence of the spike protein with mutations that maintain it in a pre-fusion conformation [24]. The poly-A tail of 100 residues has a stabilizing sequence (GCAUAUGACU) placed after the first 30 nucleotides. An experimental vaccine in trials by Moderna encodes the hemagglutinin of influenza A/Jiangxi-Donghu/346/2013 (H10N8) virus [96]. **(b) **Lipid nanoparticles are used to protect and deliver mRNA vaccines via injection into the deltoid muscle. Once inside cells, the mRNA is translated and the encoded viral protein is expressed, as well as processed into peptides and presented on MHC I molecules on the cell surface. Antigen-presenting cells can additionally internalize the expressed proteins and display viral peptides on MHC II molecules.

When considering alternative vaccine platforms for seasonal influenza, the expedited rollout of COVID-19 vaccines has propelled mRNA to the forefront of scientific and political discussion. A number of vaccine formats are in use in different countries, including two based on mRNA: Moderna’s mRNA-1273 and Pfizer-BioNTech’s BNT162b2. Both comprise messenger RNA encoding the spike protein of SARS-CoV-2 encapsulated in lipid nanoparticles. These formulations are injected intramuscularly in two doses three to 4 weeks apart [23, 24], with booster doses administered 6 months after receipt of the second dose. Additional boosters have been recommended in various countries for individuals at heightened risk of severe disease, to ensure protection is maintained as the pandemic continues [25, 26]. mRNA vaccines deliver transcripts encoding viral protein to cells, which use their own machinery to synthesize and express these proteins (Fig. 1). Viral peptides are then presented on the surface of cells and activate both humoral and cellular immune responses, mimicking a natural infection without the use of infectious virus. The SARS-CoV-2 vaccines using this method boast high efficacy and significantly reduce the frequency of severe illness and hospitalization in those who have received two doses [23, 27].

Influenza vaccines using mRNA platforms have been in development for many years. In 2012, *in vitro-*synthesized mRNA vaccines of multiple IAV subtypes were tested in mice, ferrets, and swine [28]. These vaccines were found to be immunogenic and protective against both matched and heterologous challenges. Non-human primates also responded well to mRNA vaccines delivered in lipid nanoparticles, showing sustained humoral responses at levels comparable to a licensed inactivated vaccine [29]. mRNA vaccine production and delivery technology has improved dramatically in the intervening decade; this progress has been comprehensively reviewed elsewhere [30, 31]. With the success of the SARS-CoV-2 mRNA vaccines, the possibility of a new generation of influenza vaccines based on this platform should be considered.

## Advantages of mRNA vaccines for manufacturing and distribution

### Current challenges

The constant antigenic evolution of influenza viruses necessitates annual updating of influenza vaccines. The Global Influenza Surveillance and Response System (GISRS) under the WHO collects and evaluates samples to characterize the antigenicity of prevalent circulating strains and make recommendations for strains to be included in the Northern and Southern Hemisphere vaccines. Strain recommendations are made at least 6 months in advance of the next influenza season to allow time for licensing, manufacturing, and distribution [32].

**Fig. 2.**
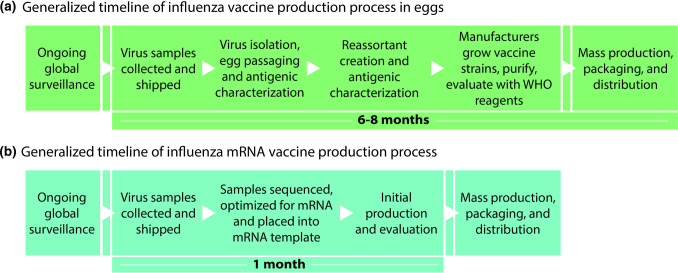
The speed of vaccine production varies dramatically between formats. a. The general timeline of the current influenza vaccine development process for inactivated vaccines, which occurs twice annually for preparation of vaccines for Northern and Southern Hemispheres. **b.** A putative timeline of mRNA-based annual influenza vaccines.

Many drawbacks of current influenza vaccination systems relate to the time required each year to select, then manufacture these updated vaccines (Fig. 2). Delays at any point in the process could become significant impediments to timely distribution and revisions are not feasible if the predicted strains do not ultimately match the circulating viruses. The interval between vaccine strain recommendation and vaccine distribution also allows time for circulating viruses to evolve, which could lead to vaccine mismatch and reduced effectiveness [33].

### A shorter timeline for production

Given the challenging timeline for annual updating, an ideal new platform for influenza vaccines would significantly reduce the time required for production. Concomitantly, such a platform would allow quicker formulation and deployment of vaccines in the event of an influenza pandemic. mRNA vaccines offer clear advantages in this regard. In response to the SARS-CoV-2 pandemic, mRNA vaccines were the first to enter clinical trials. Moderna produced an initial batch of the mRNA-1273 vaccine within a month of the viral genome sequence becoming publicly available and injected their first phase I trial participant 63 days after receipt of the sequence [34, 35]. Once manufacturers optimize an mRNA format for encoding influenza virus antigens, which includes introduction of structural features to ensure efficient translation, the protein coding sequence could be updated as needed [36]. This approach could eliminate much of the delay between strain selection and vaccine deployment [37].

Although original COVID-19 vaccines have not been updated to date, the SARS-CoV-2 pandemic may provide an example of this approach. Because available COVID-19 mRNA vaccines show reduced protection against newer variants, the idea of updating them to encode the Spike protein of currently prevalent strains is being pursued [38]. Moderna is conducting trials on a booster vaccine based on the Omicron variant, mRNA-1273.214, as well as a vaccine formula that may offer protection against a broader set of variants [39]. Policy and manufacturing hurdles encountered in this context can give insight into the feasibility of rapid-response vaccine updates to seasonal or pandemic influenza viruses.

### Geographically targeted strain selection

Rather than relying on a hemispheric system for vaccine strain selection, finer geographic resolution may also be a possibility with mRNA vaccines. Once a suitable mRNA template is developed, replacing the protein coding region would allow customization for different geographical areas. This approach could be useful in years when predominant sub-clades differ between regions of the Northern or Southern Hemisphere. An mRNA platform could also create opportunities to address heterogeneity in the timing of epidemics. Countries in the tropics frequently have outbreaks that are out of sync with their hemisphere, which poses problems for the timing of vaccine distribution and administration [40, 41]. Tailoring vaccine deployment to specific regions would be more feasible with a quicker production system and may improve effectiveness.

### The end of egg production?

Burnet’s 1936 discovery that influenza viruses could be grown in embryonated chicken eggs was a major breakthrough that laid the foundation for influenza research and vaccine development [42]. Nearly a century later, eggs are still the primary substrate for production of both inactivated and live attenuated influenza vaccines [43]. Reliance on eggs engenders multiple concerns: robust viral propagation requires that the HA and NA of circulating strains be recombined with the internal genes of a virus adapted to replicate in eggs, adding to the time and labour required for vaccine strain development. Current H3N2 seasonal strains propagate particularly poorly in eggs, which hinders their initial isolation [44, 45]. Culturing human-derived viruses in eggs can select for mutations in the HA head that affect antigenicity and can therefore result in mismatch of the vaccine product to the circulating strain [46]. Finally, this production method requires a steady supply of eggs, which can be an issue when there are wide-spread shipping delays or poultry illnesses.

**Fig. 3.**
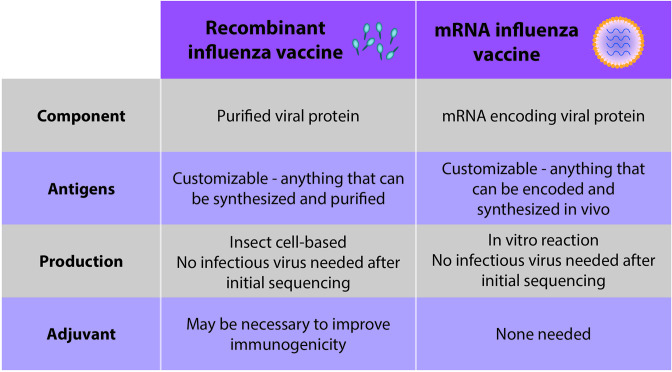
Comparison of recombinant influenza vaccines and mRNA vaccines. Recombinant influenza vaccines and mRNA vaccines share a number of advantageous features.

Cell culture-based propagation of vaccine strains is one currently-used alternative to egg-based vaccines, though yields are lower than those of eggs and cell-based vaccines can cost up to 1.5 times more than egg-based inactivated vaccines [47]. Recombinant subunit vaccines, which were first approved in the U.S. in 2013, also circumvent eggs (Fig. 3). Here, the HA protein of the vaccine strain is expressed in insect cells, with the benefit that, once the cDNA of the vaccine strain is obtained, no infectious influenza virus is involved in the process. This simplifies biosafety and eliminates the potential for the antigen to change as a result of viral evolution in the production substrate. Similarly, a switch to mRNA vaccines would eliminate the need for eggs and propagation of viruses altogether.

## Immunogen considerations for influenza mRNA vaccines

### The perennial problems of antigenic drift and shift

Current influenza vaccines are designed to elicit responses primarily to the HA protein on the surface of the virus. Selective pressure from antibodies, however, causes antigenic drift whereby the immunodominant regions of the HA and NA proteins accumulate mutations which allow immune escape [48, 49]. While H1N1 and H3N2 subtypes of IAV circulate through the human population in a recurring fashion, drifted variants of each routinely replace ancestral strains, rendering the vaccine antigens outdated.

Aside from annual seasonal outbreaks, influenza viruses have caused four pandemics in the past century. The worst of these, beginning in 1918, led to an estimated more than 50 million deaths worldwide [50]. Pandemics are caused by antigenically novel strains that can move quickly through a naïve population and are difficult to predict. Sporadic animal-to-human transmission of IAV can result in a pandemic if the virus achieves sustained inter-human transmission [51]. Responding to a newly-arisen pandemic influenza virus with an effective targeted vaccine takes time. During the 2009 influenza pandemic, the first human infections were reported in mid-April and vaccine distributions began in late September, by which time the virus had gained a foothold in more 200 countries with more than 400 000 confirmed cases globally [52]. Currently, stockpiles of vaccines against strains of concern are held in reserve for early deployment during an outbreak, but there is no guarantee they will ultimately be used [53]. Vaccines that can provide protection against a broad swath of potentially-pandemic influenza viruses, as well as seasonal strains, would allow more efficient preparation and quicker deployment.

### A universal goal

A necessary consideration when evaluating new vaccine platforms for influenza vaccination is the potential to elicit immune responses robust to antigenic change. Taking this logic further, the possibility of a ‘universal’ flu vaccine that would offer protection against all influenza viruses rather than just the seasonal strains is extremely attractive [54]. This would negate the need to match strains each season, minimize the impact of new variants on vaccine effectiveness, and allow better preparation for outbreaks since vaccine manufacturing would not be subject to the same time restraints as the current system demands. In the last decade, effectiveness of the seasonal influenza vaccines has ranged from 20–60 %, largely dependent on whether the final vaccine product matched the circulating strains [55]. Switching to an annual universal vaccine that is effective against all potential IAVs each season could prevent up to 17 million illness cases per year in the United States alone at the current vaccine uptake rate [56].

Two techniques to achieve more universal responses to influenza viruses are to use poly-valent vaccines or incorporate conserved antigens. Poly-valency is currently used in each year’s seasonal vaccines, which contain one H1N1, one H3N2 and up to two IBV strains. Increasing the number of strains could allow protection against a broader set of co-circulating viruses and is likely feasible [57]. Poly-valency is used for licensed human papilloma virus vaccines, with up to nine serotypes included in a dose [58]. However, this method is subject to the same production limitations as current formulations, which would be even more cumbersome for a larger number of strains. Protection would furthermore continue to be limited by the included strains. A promising alternative is vaccination with one or more antigens that are both immunogenic and broadly-conserved. Re-targeting vaccine-induced immunity in this way could offer protection across drifted strains, subtypes, or potentially all influenza viruses and lead to a truly universal vaccine.

### Opportunities offered by mRNA

The flexibility of the mRNA vaccine platform may allow use of alternative antigens that are more highly conserved than HA, as replacing the protein encoded in the mRNA sequence is fairly simple. Since the sequence is translated directly from the delivered mRNA within target cells, developers can make modifications to improve stability or immunogenicity. In the case of the SARS-CoV-2 spike protein encoded by the Moderna and Pfizer vaccines, there are two rigid proline residues inserted to maintain the protein in a conformation that better elicits neutralizing responses [23, 24].

There are several targets in influenza viruses that hold potential as universal antigens and could be encoded as-is or in a tailored form in a next-generation mRNA vaccine. HA and NA, while highly variable, contain regions conserved between viral strains [59]. Several studies examining whether responses to these regions can be produced with mRNA have been published over the past decade. An mRNA-lipid nanoparticle vaccine encoding full-length HA elicits responses to the stem region of HA in mice, rabbits, and ferrets after a single dose and mice were protected from heterologous influenza challenges [60]. Mice vaccinated with NA-mRNA from an H1N1 influenza virus were fully protected from lethal challenge with the homologous strain and a majority of mice survived challenge with an H5N1 virus [28].

Combining conserved antigens with polyvalency, possibly including internal viral protein antigens, is an avenue to improved protection via both humoral and cellular immunity [61–63]. Multivalent immunization using the mRNA format has been shown to confer broad cross-protection in mice even at low doses [64]. Including multiple antigenic sources in mRNA vaccines is a realistic avenue toward a more universal vaccine, though development of assays to evaluate the correlates of protection for protein targets other than HA is needed for proper determination of the effectiveness of this strategy.

mRNA vaccines also offer potentially important advantages related to the glycosylation of antigens. The glycan content of HA and NA can modulate receptor binding, mask antigenic sites, and affect immune recognition [65]. Differences in glycan patterns of vaccine antigens compared to the circulating strains have been implicated in decreased effectiveness of the vaccine due to poor virus neutralization [65–68]. Since the glycans added to HA can vary with the cell type in which the protein is expressed, production in eggs, insect cells or mammalian cells produces differentially glycosylated antigens, each of which may differ again from circulating influenza viruses [66, 69]. In the case of mRNA vaccines, protein glycosylation would occur in the vaccinated individual, eliminating species-specific glycosylation differences.

### mRNA vaccine immunogenicity

An additional factor to consider when evaluating the mRNA vaccine platform is the intrinsic immunogenicity seen in both the RNA itself and the delivery vehicles. Synthetic mRNA can trigger pattern recognition receptors within cells if recognized as originating externally, which can lead to production of Type one interferon, recruitment of immune cells, and anti-viral responses [70, 71]. This property allows mRNA to act as an adjuvant [72], precipitating a potent immune response that pulls in a wide variety of immune effectors. However, this response could also impede the development of safe and effective vaccines, as reactogenicity and the targeted destruction of mRNAs before optimal translation has occurred are possible. With knowledge of the molecular mechanisms governing innate immune responses to mRNA vaccines, their properties can be manipulated to enhance or dampen immune triggers [73, 74]. For example, immunogenicity can be modulated by altering the 5′ cap, optimizing codon usage, using modified nucleosides, controlling the potential for secondary structure formation, and shielding mRNA from pattern recognition receptors within a delivery vehicle [72].

The details of innate immune activation of the mRNA vaccines in use for SARS-CoV-2 are beginning to be understood and can inform future mRNA vaccine development [75]. Although several immunogenicity-dampening methods were used in the development of the mRNA used for these vaccines, inflammatory side-effects, from swelling at the injection site to myocarditis, have been reported [23, 24, 76–78]. Evaluation of mRNA vaccine recipients suggests that an innate response to the first dose occurs but is enhanced following the second dose [79]. Vaccine-delivered mRNA has been detected in a variety of innate cells and tissues, including the spleen, and is associated with markers consistent with mRNA immune activation [80]. Immune activation has also been linked to the lipid nanoparticle vehicle used in these vaccines [81].

In addition to offering manipulatable immunogenicity determinants, mRNA offers an attractive opportunity to improve longevity of antigen presence in the vaccinee through the use of self-amplifying mRNA vaccines. In this approach, the transcript for a replicase is included in the mRNA construct. Upon expression within target cells, the replicase produces more mRNA to prolong the expression of the viral antigens, bolstering innate and adaptive responses. This method has been pursued with a number of viruses and a self-amplifying SARS-CoV-2 mRNA vaccine is in clinical trials, which have reported positive initial data [82]. While IAV self-amplifying vaccines have not made it to trial yet, several have been reported in animal studies: a self-replicating mRNA vaccine encoding an HA from an H1N1 IAV was found to be comparably immunogenic in mice to current seasonal vaccines at low doses [83] and other self-replicating HA-mRNA vaccines have shown heterologous protection from viral challenges [84].

Adjuvanted inactivated influenza vaccines are already in use for those 65 years or older, however most current influenza vaccines are non-adjuvanted [12]. Recombinant vaccines are known to be poorly immunogenic in some populations, an issue that could potentially be addressed with the addition of adjuvants [85]. As the complexities of the intrinsic immunostimulatory properties of mRNA and lipid nanoparticles are defined, we are likely to see exciting opportunities for fine-tuning of mRNA vaccine products to achieve an optimal balance of immunogenicity and reactogenicity.

## The real-world potential of mRNA vaccines for influenza

### Early progress

A handful of pre-clinical and early clinical trials are already completed or in progress for mRNA-based influenza vaccines. A phase I trial of lipid nanoparticle mRNA vaccines encoding the full-length HA of H10N8 or H7N9 IAV, avian viruses with pandemic potential, was performed to examine safety of both intramuscular and intradermal administration [86]. The vaccines were well-tolerated and elicited high, long-lasting antibody titres, comparable to existing vaccines. The reported adverse events from that 2019 study, which included injection site pain and swelling, myalgia, and fatigue, are similar to those reported since for coronavirus mRNA vaccines [87].

Moderna has enrolled subjects in trials to test mRNA-1010, a quadrivalent seasonal formulation, and initial data from their phase I study in adults and older adults has shown it to be safe and to elicit antibody responses [88]. Similarly, Pfizer is currently enrolling subjects over age 65 for a phase I trial of mRNA vaccines for seasonal influenza. Several companies are investigating the possibility of a multivalent vaccine containing both SARS-CoV-2 and influenza virus antigens: Novavax is testing a recombinant nanoparticle vaccine and Moderna has announced that they are combining influenza and SARS-CoV-2 antigens into a single vaccine [89].

### Potential barriers

As a large proportion of those with access to mRNA vaccines received at least one dose in 2021, it may be expected that an mRNA influenza vaccine would be well-received as an alternative to the standard influenza vaccines. Vaccine hesitancy and disinformation have become hurdles, however, in the endeavour to achieve herd immunity against SARS-CoV-2. In the United States, politicization of public health protocols and partisan uptake discrepancies have hampered pandemic control [90, 91]. Poor vaccine perception and hesitancy has impacted deployment in many countries, even when vaccine supply is ample, leading to uneven coverage within countries and across geographical regions. Polarized vaccine acceptance may impact public perception of future mRNA formulations for viruses other than SARS-CoV-2.

Deploying mRNA vaccines globally will also present obstacles. A stark divide appeared during the distribution of coronavirus vaccines, with some high-income countries like Canada and Spain attaining vaccination rates in excess of 80 % of their total population while some low-income countries had not reached 10 % by January of 2022 [92]. Influenza vaccines also show a distribution disparity, with 95 % distributed within the Americas, Europe, and the West Pacific in 2015, regions that are home to only 50% of the global population [93]. Developing a vaccine system that is cheap, safe, quick, and easily transportable may alleviate some of these inequalities. mRNA vaccines have the potential to check many of those boxes for more accessible and equitable distribution, particularly in ease of production and affordability. A pitfall that still needs to be resolved, however, is the need for cold storage of mRNA vaccines. Current coronavirus vaccines must remain frozen during transport and storage, which impedes dissemination to rural areas; however, as stability data have become available, storage requirements have become less stringent [94, 95]. Nevertheless, storage innovations are necessary to provide broader access and are potentially attainable, as a lyophilized influenza mRNA vaccine was shown to remain stable at 37 °C for several weeks [28]. Current seasonal influenza vaccines require refrigeration but eliminating the need for a cold chain altogether is necessary to distribute vaccines equitably to rural and low-income areas.

### The road ahead

While mRNA vaccines for influenza have been under development for many years, the success of the SARS-CoV-2 mRNA vaccines has brought their clinical use to the forefront of possibility. The ease of producing large quantities of custom, well-controlled vaccines in a relatively short time span is attractive compared with the current vaccine platforms. Many of the regulatory questions and logistical considerations that might arise from deploying a new technology are being confronted during the roll-out of the coronavirus vaccines, which should pave a smoother road for next-generation influenza vaccines in the near future.

## References

[1] Barberis I, Myles P, Ault SK, Bragazzi NL, Martini M (2016). History and evolution of influenza control through vaccination: from the first monovalent vaccine to universal vaccines. J Prev Med Hyg.

[2] Francis T (1953). Vaccination against influenza. Bull World Health Organ.

[3] Plotkin SA, Orenstein WA, Offit PA (2018). Plotkin’s Vaccines.

[4] Palese P, Racaniello VR, Desselberger U, Young J, Baez M (1980). Genetic structure and genetic variation of influenza viruses. Philos Trans R Soc Lond B Biol Sci.

[5] Javanian M, Barary M, Ghebrehewet S, Koppolu V, Vasigala V (2021). A brief review of influenza virus infection. J Med Virol.

[6] Gamblin SJ, Skehel JJ (2010). Influenza hemagglutinin and neuraminidase membrane glycoproteins. J Biol Chem.

[7] Gamblin SJ, Vachieri SG, Xiong X, Zhang J, Martin SR (2021). Hemagglutinin structure and activities. Cold Spring Harb Perspect Med.

[8] Lai JCC, Chan WWL, Kien F, Nicholls JM, Peiris JSM (2010). Formation of virus-like particles from human cell lines exclusively expressing influenza neuraminidase. J Gen Virol.

[9] Mostafa A, Abdelwhab EM, Mettenleiter TC, Pleschka S (2018). Zoonotic potential of influenza A viruses: a comprehensive overview. Viruses.

[10] Keshavarz M, Mirzaei H, Salemi M, Momeni F, Mousavi MJ (2019). Influenza vaccine: Where are we and where do we go?. Rev Med Virol.

[11] (CDC) CfDCaP (2021). Different Types of Flu Vaccines. https://www.cdc.gov/flu/prevent/different-flu-vaccines.htm.

[12] Del Giudice G, Rappuoli R (2015). Inactivated and adjuvanted influenza vaccines. Curr Top Microbiol Immunol.

[13] Jin H, Subbarao K (2015). Live attenuated influenza vaccine. Curr Top Microbiol Immunol.

[14] Maassab HF, Bryant ML (1999). The development of live attenuated cold-adapted influenza virus vaccine for humans. Rev Med Virol.

[15] Paget J, Spreeuwenberg P, Charu V, Taylor RJ, Iuliano AD (2019). Global mortality associated with seasonal influenza epidemics: New burden estimates and predictors from the GLaMOR Project. J Glob Health.

[16] Organization WH (2018). Influenza (Seasonal). https://www.who.int/news-room/fact-sheets/detail/influenza-(seasonal).

[17] OECD (2021). Influenza vaccination rates (indicator).

[18] Iuliano AD, Roguski KM, Chang HH, Muscatello DJ, Palekar R (2018). Estimates of global seasonal influenza-associated respiratory mortality: a modelling study. Lancet.

[19] Osterholm MT, Kelley NS, Sommer A, Belongia EA (2012). Efficacy and effectiveness of influenza vaccines: a systematic review and meta-analysis. Lancet Infect Dis.

[20] Bartley JM, Cadar AN, Martin DE (2021). Better, Faster, Stronger: mRNA Vaccines Show Promise for Influenza Vaccination in Older Adults. Immunol Invest.

[21] Hughes K, Middleton DB, Nowalk MP, Balasubramani GK, Martin ET (2021). Effectiveness of Influenza Vaccine for Preventing Laboratory-Confirmed Influenza Hospitalizations in Immunocompromised Adults. Clin Infect Dis.

[22] Organization GWH (2019-2030). Global influenza strategy.

[23] Baden LR, El Sahly HM, Essink B, Kotloff K, Frey S (2021). Efficacy and Safety of the mRNA-1273 SARS-CoV-2 Vaccine. N Engl J Med.

[24] Polack FP, Thomas SJ, Kitchin N, Absalon J, Gurtman A (2020). Safety and Efficacy of the BNT162b2 mRNA Covid-19 Vaccine. N Engl J Med.

[25] Health IMo (2022). Covid-19 Vaccines for 12-year-olds and Older. https://corona.health.gov.il/en/vaccine-for-covid/over-12/.

[26] (CDC) CfDCaP (2022). Stay Up to Date with Your COVID-19 Vaccines. https://www.cdc.gov/coronavirus/2019-ncov/vaccines/stay-up-to-date.html.

[27] Tregoning JS, Flight KE, Higham SL, Wang Z, Pierce BF (2021). Progress of the COVID-19 vaccine effort: viruses, vaccines and variants versus efficacy, effectiveness and escape. Nat Rev Immunol.

[28] Petsch B, Schnee M, Vogel AB, Lange E, Hoffmann B (2012). Protective efficacy of in vitro synthesized, specific mRNA vaccines against influenza A virus infection. Nat Biotechnol.

[29] Lutz J, Lazzaro S, Habbeddine M, Schmidt KE, Baumhof P (2017). Unmodified mRNA in LNPs constitutes a competitive technology for prophylactic vaccines. NPJ Vaccines.

[30] Pardi N, Hogan MJ, Porter FW, Weissman D (2018). mRNA vaccines - a new era in vaccinology. Nat Rev Drug Discov.

[31] Pilkington EH, Suys EJA, Trevaskis NL, Wheatley AK, Zukancic D (2021). From influenza to COVID-19: Lipid nanoparticle mRNA vaccines at the frontiers of infectious diseases. Acta Biomater.

[32] Chen J-R, Liu Y-M, Tseng Y-C, Ma C (2020). Better influenza vaccines: an industry perspective. J Biomed Sci.

[33] Kim H, Webster RG, Webby RJ (2018). Influenza Virus: Dealing with a Drifting and Shifting Pathogen. Viral Immunol.

[34] (2020). Moderna ships mRNA vaccine against novel coronavirus (mRNA-1273) for phase 1 study [Press Release]. 02/24/2020 2020.

[35] (2020). Moderna announces first participant dosed in NIH-led phase 1 study of mRNA vaccine (mRNA-1273) against novel coronavirus [Press Release]. 03/16/2020.

[36] Dormitzer PR (2015). Rapid production of synthetic influenza vaccines. Curr Top Microbiol Immunol.

[37] To KKW, Cho WCS (2021). An overview of rational design of mRNA-based therapeutics and vaccines. Expert Opin Drug Discov.

[38] Andrews N, Stowe J, Kirsebom F, Toffa S, Rickeard T (2022). Covid-19 Vaccine Effectiveness against the Omicron (B.1.1.529) Variant. N Engl J Med.

[39] Su H, Zhao Y, Zheng L, Wang S, Shi H (2020). Effect of the selection pressure of vaccine antibodies on evolution of H9N2 avian influenza virus in chickens. AMB Express.

[40] Alonso WJ, Yu C, Viboud C, Richard SA, Schuck-Paim C (2015). A global map of hemispheric influenza vaccine recommendations based on local patterns of viral circulation. Sci Rep.

[41] Dave K, Lee PC (2019). Global geographical and temporal patterns of seasonal influenza and associated climatic factors. Epidemiol Rev.

[42] Burnet FM (1936). Immunological studies with the virus of infectious laryngotracheitis of fowls using the developing egg technique. J Exp Med.

[43] Trombetta CM, Marchi S, Manini I, Lazzeri G, Montomoli E (2019). Challenges in the development of egg-independent vaccines for influenza. Expert Rev Vaccines.

[44] Stevens J, Chen L-M, Carney PJ, Garten R, Foust A (2010). Receptor specificity of influenza A H3N2 viruses isolated in mammalian cells and embryonated chicken eggs. J Virol.

[45] Lin YP, Xiong X, Wharton SA, Martin SR, Coombs PJ (2012). Evolution of the receptor binding properties of the influenza A(H3N2) hemagglutinin. Proc Natl Acad Sci U S A.

[46] Nakowitsch S, Waltenberger AM, Wressnigg N, Ferstl N, Triendl A (2014). Egg- or cell culture-derived hemagglutinin mutations impair virus stability and antigen content of inactivated influenza vaccines. Biotechnol J.

[47] (CDC) CfDCaP (2022). CDC Vaccine Price List 2022. https://www.cdc.gov/vaccines/programs/vfc/awardees/vaccine-management/price-list/index.html.

[48] Bouvier NM, Palese P (2008). The biology of influenza viruses. Vaccine.

[49] Wu NC, Wilson IA (2020). Influenza hemagglutinin structures and antibody recognition. Cold Spring Harb Perspect Med.

[50] (CDC) CfDCaP (2019). 1918 Pandemic (H1N1 virus). https://www.cdc.gov/flu/pandemic-resources/1918-pandemic-h1n1.html.

[51] (CDC) CfDCaP (2013). Emergence of avian influenza A(H7N9) virus causing severe human illness - China, February-April 2013. morbidity and mortality weekly report.

[52] Geneva WHO (2009). Pandemic influenza A (H1N1) 2009 virus vaccine - conclusions and recommendations from the october 2009 meeting of the immunization strategic advisory group of experts. Weekly Epidemiological Report.

[53] U.S. Department of Health and Human Services (2017). Pandemic Influenza Plan 2017 Update.

[54] Krammer F, García-Sastre A, Palese P (2018). Is It Possible to Develop a “Universal” Influenza Virus Vaccine? Potential Target Antigens and Critical Aspects for a Universal Influenza Vaccine. Cold Spring Harb Perspect Biol.

[55] (CDC) CfDCaP (2021). Past Seasons Vaccine Effectiveness Estimates. https://www.cdc.gov/flu/vaccines-work/past-seasons-estimates.html.

[56] Sah P, Alfaro-Murillo JA, Fitzpatrick MC, Neuzil KM, Meyers LA (2019). Future epidemiological and economic impacts of universal influenza vaccines. Proc Natl Acad Sci U S A.

[57] Worby CJ, Wallinga J, Lipsitch M, Goldstein E (2017). Population effect of influenza vaccination under co-circulation of non-vaccine variants and the case for A bivalent A/H3N2 vaccine component. Epidemics.

[58] Schlingmann B, Castiglia KR, Stobart CC, Moore ML (2018). Polyvalent vaccines: High-maintenance heroes. PLoS Pathog.

[59] Estrada LD, Schultz-Cherry S (2019). Development of a Universal Influenza Vaccine. J Immunol.

[60] Pardi N, Parkhouse K, Kirkpatrick E, McMahon M, Zost SJ (2018). Nucleoside-modified mRNA immunization elicits influenza virus hemagglutinin stalk-specific antibodies. Nat Commun.

[61] Bazhan S, Antonets D, Starostina E, Ilyicheva T, Kaplina O (2020). Immunogenicity and Protective Efficacy of Influenza A DNA Vaccines Encoding Artificial Antigens Based on Conservative Hemagglutinin Stem Region and M2 Protein in Mice. Vaccines (Basel).

[62] Starostina EV, Sharabrin SV, Antropov DN, Stepanov GA, Shevelev GY (2021). Construction and Immunogenicity of Modified mRNA-Vaccine Variants Encoding Influenza Virus Antigens. Vaccines (Basel).

[63] Zykova AA, Blokhina EA, Stepanova LA, Shuklina MA, Tsybalova LM (2022). Nanoparticles based on artificial self-assembling peptide and displaying M2e peptide and stalk HA epitopes of influenza A virus induce potent humoral and T-cell responses and protect against the viral infection. Nanomedicine.

[64] Freyn AW, Ramos da Silva J, Rosado VC, Bliss CM, Pine M (2020). A Multi-Targeting, Nucleoside-Modified mRNA Influenza Virus Vaccine Provides Broad Protection in Mice. Mol Ther.

[65] Alymova IV, York IA, Air GM, Cipollo JF, Gulati S (2016). Glycosylation changes in the globular head of H3N2 influenza hemagglutinin modulate receptor binding without affecting virus virulence. Sci Rep.

[66] An Y, Parsons LM, Jankowska E, Melnyk D, Joshi M (2019). *N*-Glycosylation of Seasonal Influenza Vaccine Hemagglutinins: Implication for Potency Testing and Immune Processing. J Virol.

[67] Vigerust DJ, Shepherd VL (2007). Virus glycosylation: role in virulence and immune interactions. Trends Microbiol.

[68] Abe Y, Takashita E, Sugawara K, Matsuzaki Y, Muraki Y (2004). Effect of the addition of oligosaccharides on the biological activities and antigenicity of influenza A/H3N2 virus hemagglutinin. J Virol.

[69] Chen W, Zhong Y, Su R, Qi H, Deng W (2017). N-glycan profiles in H9N2 avian influenza viruses from chicken eggs and human embryonic lung fibroblast cells. J Virol Methods.

[70] Edwards DK, Jasny E, Yoon H, Horscroft N, Schanen B (2017). Adjuvant effects of a sequence-engineered mRNA vaccine: translational profiling demonstrates similar human and murine innate response. J Transl Med.

[71] de Oliveira Mann CC, Hornung V (2021). Molecular mechanisms of nonself nucleic acid recognition by the innate immune system. Eur J Immunol.

[72] Linares-Fernández S, Lacroix C, Exposito J-Y, Verrier B (2020). Tailoring mRNA Vaccine to Balance Innate/Adaptive Immune Response. Trends Mol Med.

[73] Karikó K, Buckstein M, Ni H, Weissman D (2005). Suppression of RNA recognition by Toll-like receptors: the impact of nucleoside modification and the evolutionary origin of RNA. Immunity.

[74] Karikó K, Muramatsu H, Ludwig J, Weissman D (2011). Generating the optimal mRNA for therapy: HPLC purification eliminates immune activation and improves translation of nucleoside-modified, protein-encoding mRNA. Nucleic Acids Res.

[75] Fang E, Liu X, Li M, Zhang Z, Song L (2022). Advances in COVID-19 mRNA vaccine development. Signal Transduct Target Ther.

[76] Gargano JW, Wallace M, Hadler SC, Langley G, Su JR (2021). Use of mRNA COVID-19 Vaccine After Reports of Myocarditis Among Vaccine Recipients: Update from the Advisory Committee on Immunization Practices - United States, June 2021. MMWR Morb Mortal Wkly Rep.

[77] Cai C, Peng Y, Shen E, Huang Q, Chen Y (2021). A comprehensive analysis of the efficacy and safety of COVID-19 vaccines. Mol Ther.

[78] Barda N, Dagan N, Ben-Shlomo Y, Kepten E, Waxman J (2021). Safety of the BNT162b2 mRNA Covid-19 Vaccine in a Nationwide Setting. N Engl J Med.

[79] Arunachalam PS, Scott MKD, Hagan T, Li C, Feng Y (2021). Systems vaccinology of the BNT162b2 mRNA vaccine in humans. Nature.

[80] Li C, Lee A, Grigoryan L, Arunachalam PS, Scott MKD (2022). Mechanisms of innate and adaptive immunity to the Pfizer-BioNTech BNT162b2 vaccine. Nat Immunol.

[81] Tahtinen S, Tong A-J, Himmels P, Oh J, Paler-Martinez A (2022). IL-1 and IL-1ra are key regulators of the inflammatory response to RNA vaccines. Nat Immunol.

[82] (2022). Arcturus announces self-amplifying COVID-19 mrna vaccine candidate ARCT-154 meets primary efficacy endpoint in phase 3 study [press release]. 4/20/22.

[83] Hekele A, Bertholet S, Archer J, Gibson DG, Palladino G (2013). Rapidly produced SAM(®) vaccine against H7N9 influenza is immunogenic in mice. Emerg Microbes Infect.

[84] Brazzoli M, Magini D, Bonci A, Buccato S, Giovani C (2016). Induction of Broad-Based Immunity and Protective Efficacy by Self-amplifying mRNA Vaccines Encoding Influenza Virus Hemagglutinin. J Virol.

[85] Sedova ES, Shcherbinin DN, Migunov AI, Smirnov IA, Logunov DI (2012). Recombinant influenza vaccines. Acta Naturae.

[86] Feldman RA, Fuhr R, Smolenov I, Mick Ribeiro A, Panther L (2019). mRNA vaccines against H10N8 and H7N9 influenza viruses of pandemic potential are immunogenic and well tolerated in healthy adults in phase 1 randomized clinical trials. Vaccine.

[87] Ebinger JE, Lan R, Sun N, Wu M, Joung S (2021). Symptomology following mRNA vaccination against SARS-CoV-2. Prev Med.

[88] (2021). Moderna announces positive interim phase 1 data for mrna flu vaccine and provides program update [press release].

[89] Massare MJ, Patel N, Zhou B, Maciejewski S, Flores R (2021). Combination respiratory vaccine containing recombinant SARS-cov-2 spike and quadrivalentseasonal influenza hemagglutinin nanoparticles with matrix-M adjuvant. bioRxiv.

[90] Karlsson LC, Soveri A, Lewandowsky S, Karlsson L, Karlsson H (2022). The behavioral immune system and vaccination intentions during the coronavirus pandemic. Pers Individ Dif.

[91] Bokemper SE, Gerber AS, Omer SB, Huber GA (2021). Persuading US White evangelicals to vaccinate for COVID-19: Testing message effectiveness in fall 2020 and spring 2021. Proc Natl Acad Sci U S A.

[92] Ritchie HM, Edouard; Rodés-Guirao, Luis; Appel, Cameron; Giattino, Charlie; Ortiz-Ospina, Esteban; Hasell, Joe; Macdonald, Bobbie; Beltekian, Diana; Roser, Max (2020). Coronavirus Pandemic (COVID-19) [Online resource]. OurWorldInData.org202. https://ourworldindata.org/coronavirus.

[93] Ortiz JR, Neuzil KM (2019). Influenza Immunization in Low- and Middle-Income Countries: Preparing for Next-Generation Influenza Vaccines. J Infect Dis.

[94] Moderna I (2022). Fact sheet for healthcare providers administering vaccine (vaccination providers).

[95] Pfizer (2022). Manufacturing and Distributing the COVID-19 Vaccine. https://www.pfizer.com/science/coronavirus/vaccine/manufacturing-and-distribution.

[96] Bahl K, Senn JJ, Yuzhakov O, Bulychev A, Brito LA (2017). Preclinical and Clinical Demonstration of Immunogenicity by mRNA Vaccines against H10N8 and H7N9 Influenza Viruses. Mol Ther.

